# New Method of Extracting Nerves from Teeth

**Published:** 1851-01

**Authors:** M. D. Stoneman


NEW METHOD OF EXTRACTING NERVES FROM TEETH.—In this section there is now an excitement on the subject of extracting the nerve from teeth, "which is done" in the following manner: A "*horn*" made of tin, the small end curved—the curved point being held upon the sensitive part of the tooth by the patient. A small iron plate heated to redness is placed under the funnel part of the horn. In the hot iron plate or cup is placed a *pill*, the size of a common marble, prepared in the following manner, viz.: to any quantity of beeswax put in a sufficient quantity of onion seed, so that there will be 5 or 6 seeds in each *pill*. Stir the wax and seed together in a heated vessel for a few minutes, then form the pills; roll the pills in a mixture of "*soot*" and spirits of turpentine, say enough of the latter to moisten the former until well coated, when they are ready for use as above.

The heat of the *dish* under the *horn* melts the wax, and soon causes one or more of the seeds to burst with an explosive sound, on which the operator removes the *horn*, when lo! there sticks the "nerve," looking like a roasted skipper, sure enough. The patient is convinced and cured instanter. I have seen Odontalgia cured in one minute and less; it has uow become quite a business into which every one who wishes, embarks, sure of success in his own mind. Nothing more common than to see persons here ordering their horns by the dozen. A day or two since I saw them operating, and having just extracted a superior molar from a lady, which had been aching. I proposed to show them a nerve in situ; I then removed the carious bone down to the pulp, and after they had seen it fairly exposed I proposed that they operate on that tooth and *extract* the *nerve*, which was immediately done, and with their usual success, removing three or four "nerves;" I then called for a hammer and broke the tooth, showing the contents of its pulp cavities unaffected by the operation.

I make no comment on the above.

Please excuse hurry,

M. D. STONEMAN.

---

The above, by our friend, Dr. Stoneman, we give, although perhaps, not intended for publication. It describes more in full what we gave in a former number of the Register, as the introduction of steam into the practice of dentistry. The recipe for the pills is rich. We cannot decide whether the relief is obtained by the remedial agency of the pills or by the sudden fright of the patient. We have heard before of maggots being found in hollow teeth. Query—do they ever grow there?—Ed.

